# Metabolic reprogramming by miRNAs in the tumor microenvironment: Focused on immunometabolism

**DOI:** 10.3389/fonc.2022.1042196

**Published:** 2022-11-22

**Authors:** Shadia Hamoud Alshahrani, Yousif Saleh Ibrahim, Abduladheem Turki Jalil, Abdelgadir Alamin Altoum, Harun Achmad, Rahman S. Zabibah, Gamal A. Gabr, Andrés Alexis Ramírez-Coronel, Ameer A. Alameri, Qutaiba A. Qasim, Sajad Karampoor, Rasoul Mirzaei

**Affiliations:** ^1^ Medical Surgical Nursing Department, King Khalid University, Almahala, Khamis Mushate, Saudi Arabia; ^2^ Department of Medical Laboratory Techniques, Al-maarif University College, Ramadi, Al-Anbar, Iraq; ^3^ Medical Laboratories Techniques Department, Al-Mustaqbal University College, Babylon, Hilla, Iraq; ^4^ Department of Medical Laboratory Sciences, College of Health Sciences, Gulf Medical University, Ajman, United Arab Emirates; ^5^ Department of Pediatric Dentistry, Faculty of Dentistry, Hasanuddin University, Makassar, Indonesia; ^6^ Medical Laboratory Technology Department, College of Medical Technology, The Islamic University, Najaf, Iraq; ^7^ Department of Pharmacology and Toxicology, College of Pharmacy, Prince Sattam Bin Abdulaziz University, Al-Kharj, Saudi Arabia; ^8^ Agricultural Genetic Engineering Research Institute (AGERI), Agricultural Research Center, Giza, Egypt; ^9^ Health and Behavior Research Group (HBR), Catholic University of Cuenca, Cuenca, Ecuador; ^10^ Laboratory of Psychometry and Ethology, Catholic University of Cuenca, Cuenca, Ecuador; ^11^ Epidemiology and Biostatistics Research Group, Universidad CES, Medellin, Colombia; ^12^ Department of Chemistry, University of Babylon, Babylon, Iraq; ^13^ College of Pharmacy, Al-Ayen University, Thi-Qar, Iraq; ^14^ Gastrointestinal and Liver Diseases Research Center, Iran University of Medical Sciences, Tehran, Iran; ^15^ Venom and Biotherapeutics Molecules Lab, Medical Biotechnology Department, Biotechnology Research Center, Pasteur Institute of Iran, Tehran, Iran

**Keywords:** MicroRNAs, cancer, metabolism, immunometabolism, immune cell

## Abstract

MicroRNAs (miRNAs) are emerging as a significant modulator of immunity, and their abnormal expression/activity has been linked to numerous human disorders, such as cancer. It is now known that miRNAs potentially modulate the production of several metabolic processes in tumor-associated immune cells and indirectly *via* different metabolic enzymes that affect tumor-associated signaling cascades. For instance, Let-7 has been identified as a crucial modulator for the long-lasting survival of CD8^+^ T cells (naive phenotypes) in cancer by altering their metabolism. Furthermore, in T cells, it has been found that enhancer of zeste homolog 2 (EZH2) expression is controlled *via* glycolytic metabolism through miRNAs in patients with ovarian cancer. On the other hand, immunometabolism has shown us that cellular metabolic reactions and processes not only generate ATP and biosynthetic intermediates but also modulate the immune system and inflammatory processes. Based on recent studies, new and encouraging approaches to cancer involving the modification of miRNAs in immune cell metabolism are currently being investigated, providing insight into promising targets for therapeutic strategies based on the pivotal role of immunometabolism in cancer. Throughout this overview, we explore and describe the significance of miRNAs in cancer and immune cell metabolism.

## Introduction

Metabolism is an energy production mechanism that cells use to maintain cellular balance, proliferation, and differentiation (1). Aerobic glycolysis, known as the Warburg effect, is a distinctive metabolic feature of tumor metabolism (2). Cancer cells, unlike normal cells, generate most of their energy through increased rates of glycolysis in the cytosol rather than pyruvate oxidation in the mitochondria (3). Over the last decade, there has been a growing emphasis on immunometabolism, or the function of metabolism in stimulating and controlling immune cells (4, 5). Due to the apparent essential role of intracellular metabolism in a set of cellular and biochemical processes to obtain and use nutrients, including lipids, proteins, and carbohydrates, to generate energy *via* adenosine triphosphate (ATP), as well as other biomolecules, it is not surprising that energy generation *via* cellular metabolism would be a significant determinant of the destiny and activity of immune cells (4, 6). Furthermore, aberrant cellular metabolism leads to immune cell malfunction, which is frequently connected with immunological-related diseases, such as cancer (7–9).

miRNAs are endogenous (~22nt) RNAs that can control many biological functions (10). Compelling evidence suggests that miRNAs play an essential part in energy metabolism, notably lipid and glucose metabolism and also amino acid biosynthesis (11). Furthermore, miRNAs may identify and modify metabolic components at the transcriptional level, which is essential in both non-neoplastic and tumor cells (12). For instance, miR-143 controls glycolysis by affecting Hexokinase 2 (HK2), which phosphorylates glucose to generate Glucose 6-phosphate (G6P), therefore allocating glucose to the glycolytic process. Malignant cell metabolism might also be changed to avoid apoptosis and promote cell proliferation and survival. The Warburg effect, in which miRNA dysregulation leads to higher glycolysis, is the best-characterized metabolic phenotype found in cancer cells (13, 14). However, a growing body of evidence suggests that miRNAs are also heavily engaged in the metabolic regulation of immune cells in cancer. miRNAs seem to have an impact on the Phosphoinositide 3-kinase (PI3K)/protein kinase B (AKT) network, which is a signaling system that regulates glucose metabolism *via* reducing phosphatase and tensin homolog (PTEN) activities, which might increase the expression of interleukin (IL)-4, IL-10, transforming growth factor-beta (TGF-β), and arginase, and found in the inflammatory zone (FIZZ) in M2 macrophages (15–17). This review presents recent results on the action of miRNAs in cancer metabolism, focusing on immune cells.

## Immunometabolism at a glance

Cellular metabolism is a complex metabolic process that turns metabolites into biological activity (18). Catabolic pathways at the center of this physiological network decompose substances to create energy, which is subsequently utilized to fuel biochemical mechanisms and perform mechanical activity. Metabolism generates energy for cellular operations by producing ATP (19). Metabolism decomposes nutrients like protein, sugar, and fat into smaller units like glucose, fatty acids, and amino acids. This procedure has the potential to produce energy (19). Metabolism transforms simpler components like lipids, proteins, and nucleotides into macromolecules (i.e., anabolism). Energy is required for this procedure. Furthermore, metabolism involves cellular tasks other than anabolism, catabolism, and energy, such as cellular signaling and gene transcription (19). Metabolites, for instance, act as precursors for posttranslational protein modification to induce changes in protein functionality or control epigenetics to induce alterations in gene expression.

Immunometabolism, or the change in metabolic processes and activity of immune cells, is a new field of study that has gained international interest in the last decade (20). Metabolic reprogramming leads to variations in biosynthetic processes and metabolite quantities that occur throughout the proliferation and differentiation (21). Metabolic rewiring occurs in all cell types, including cancer and immunological cells (21). Because they govern downstream transcriptional and posttranscriptional processes, these modifications are crucial for a proper immune reaction, and their deregulation may impair growth, effector functions, and propagation (22). In summary, immune cells will increase the expression of aerobic glycolysis in an inflammatory setting (as observed in B cells, effector T helper type 1 (Th1), T helper 17 (Th17) cells, natural killer (NK) cells, dendritic cells, and M1 macrophages), whereas metabolism based on oxidative phosphorylation (OXPHOS) typically facilitates an anti-inflammatory characteristic (as detected in regulatory T (Treg) cells and M2 macrophages) (22). The metabolic rate of resting or naive cells is normally low in the form of low glycolysis and OXPHOS (22). M0 macrophages differentiate into M1 and M2 phenotypes, while naïve CD4+ T cells become effector T cells or Tregs, and quiescent NK cells become activated. In fact, each cell subtype has distinct metabolic requirements and cytokine repertoires (22). There will be much to learn about metabolic and immune control in future research into disease states. In addition to systemic metabolic alterations, innate metabolic changes are related to building an immune reaction and functional programming inside a cell (23). One of the most prominent of these changes is the stimulation of anaerobic glycolysis, a common characteristic of T cells, dendritic cells (DCs), and macrophages involved in proinflammatory stimulation (24–26).

## The role of metabolic pathways in cancer cells

The Warburg effect is tumor cells’ most well-known metabolic switching mechanism (27). Compared to normal cells, cancer cells prefer glycolysis to mitochondrial OXPHOS, even when oxygen levels are high (28). Since glycolysis is inefficient for ATP synthesis, its output is considerably faster, supplying energy to cancer cells for growth and proliferation. Moreover, glycolysis provides tumor cells with various building components for biomass production (29). The oncogene c-MYC and the hypoxia-inducible factor-1 (HIF-1) facilitate the development of essential enzymes that boost aerobic glycolysis, the most significant of which are HK2, pyruvate kinase 2 (PKM2), glucose transporter 1 (GLUT1), and lactate dehydrogenase A (LDHA) (28–30). The upregulation of GLUT1 promotes glucose absorption by tumor cells. An increase in the expression of HK2 converts glucose into G6P, the first step in glycolysis, and elevates its flow to the pentose phosphate pathway (PPP), causing the formation of nicotinamide adenine dinucleotide phosphate oxidase (NADPH). NADPH is required for anaerobic activities, such as nucleotide synthesis, as well as body defense against reactive oxygen species (ROS) (28, 29).

Many studies have shown comparable increases in glutamine uptake by tumor cells as well as improvements in the tricarboxylic acid (TCA) cycle, PPP, lipid catabolism, and other metabolic activities observed in rapidly proliferating normal cells (31). In this context, the primary distinction between tumor and propagating normal cells is that the latter’s intake of fuel molecules is rigorously managed by growth factor signaling (32). On the other hand, tumor cells absorb glucose and glutamine from the extracellular media due to mutations in key metabolic regulatory genes, including c-MYC, AKT, and PI3K (31). Modifications in the function of these and other genes cause cell-autonomous increases in the abundance and the function of membrane transporters and metabolic enzymes, allowing for increased glycolysis and glutaminolysis in the absence of external stimuli (33). This allows efficient coupling of energy and biosynthesis requirements for uncontrolled cell growth.

## Glucose metabolism of tumor cells

However, in certain tumor cells, even in the presence of oxygen, glucose enters the cytoplasm and performs a chemical process catalyzed by lactate dehydrogenase and pyruvate kinase to create lactate and nicotinamide adenine dinucleotide + (NAD+) rather than entering the tricarboxylic acid (TCA) cycle, as is the case in normal cells (34, 35). Compared to glycolysis, the TCA cycle generates almost 20 times the amount of energy from a single glucose molecule (36). In contrast to the mitochondrial TCA cycle, glycolysis may simultaneously convert one glucose molecule into 10-100 times as much ATP (2). This efficient strategy may be selected by tumor cells because glycolysis improves tumor cell competitiveness in a nutrient-poor niche (2). Furthermore, the pentose phosphate pathway (PPP) (another branch of glycolysis) can be used to generate pentose. In this process, neither ATP nor any other form of cellular energy is produced or utilized. G-6-P dehydrogenase (G6PDH) and 6-phosphogluconate dehydrogenase (6PGDH) accelerate the conversion of glucose-6-phosphate (G-6-P), a byproduct of glycolysis into nicotinamide adenine dinucleotide phosphate (NADPH) and ribose-5-phosphate (R-5-P) (37). Glycolysis may restart using R-5-P when it is converted back into fructose-6-phosphate (F-6-P) and glyceraldehyde-3-phosphate (G-3-P). One of the universal electron carriers, NADPH, includes minimizing intracellular oxidation by controlling the transformation of oxidized glutathione to decreased glutathione and interacting with a broad range of enzymes. It accomplishes this by transporting electrons and hydrogen released by the energy of sunlight (38). Reactive oxygen species (ROS) and other oxides are byproducts of several cellular metabolic activities; they attach to proteins and are degraded in the presence of NADPH, which maintains the function of these proteins (38). Additionally, R-5-P generated by PPP may give a rapid rate of synthesis of nucleic acid for cancerous cells (38).

## Glutamine metabolism of tumor cells

A metabolic fuel, glutamine enables rapidly replicating cells to fulfill their increasing demands for ATP, biochemical precursors, and reductants (39). Solute carrier family 1 member 5 (SLC1A5) is responsible for transporting glutamine into the cell; GLS then catalyzes the dehydrogenation process in the mitochondria to produce glutamate. GLUD, alanine, or aspartate transaminase (TAs) transform glutamate to -KG, a byproduct of the TCA cycle (40). Furthermore, signaling molecules such as RAS, MP-activated protein kinase (AMPK), and protein kinase B (PKB) A activate glycolytic enzymes and drive lactate generation, forcing tumor cells to consume glutamine to fulfill their enhanced energy demands (41). In addition to supplying energy, glutamine is essential for tumor cell biogenesis. When glutamine reaches the cell, the activity of GLS and GLUD produces ammonia, which may be employed immediately in the production of purines and pyrimidines (42). It has since been discovered that glutamine creates -KG, a type of raw material used in the production of purines and pyrimidines, *via* a redox interaction with aspartic acid (43, 44). Glutamate catabolism in tumor cells promotes the production of nucleotide precursors, which aids in tumorigenesis and replication. Glutamine also serves as a nitrogen source in protein production; isotope tracking indicated that glutamine supplies almost half of the non-essential amino acids needed by cancer cells for protein biosynthesis (40, 42). Finally, in hypoxic circumstances, cancer cells may utilize glutamine to create citric acid and fatty acids *via* reducing carboxylation processes while also constructing dihydro-orotate acid to alleviate the negative consequences of ammonia on cancer cells (45, 46).

## Immune cell metabolism

The highly active metabolic processes of tumor cells can cause significant differences in the form of nutrients and other small molecules in the tumor microenvironment (TME) (47). This can have severe consequences for the immune system. Increased metabolic activities of cancer cells and impaired angiogenesis within the TME may lead to nutrient deficiency as well as a hypoxic environment, leading to metabolic competition between tumor cells and invading immune cells (48–50). Similarly, in animal models, glucose uptake and the effective function of anti-tumor CD4^+^ T cells are inversely related to the glycolytic activity of tumor cells, and access to glucose in the TME increases cytokine production from anti-tumor CD8^+^ T cells (48). The key effector component of the antitumor response is composed of CD4^+^ T cells (CD4^+^ Conv) and CD8^+^ effector T cells (Teff), while non-proliferative naive CD4^+^ and CD8^+^ T cells identify their relevant antigens in the setting of co­stimulatory signaling. In fact, they become proliferate and activate metabolic characteristics to facilitate rapid expansion (51–53). Even though many early studies identified increased aerobic glycolysis as a characteristic of T cell activation, it is now obvious that increased TCA cycle metabolism, as well as OXPHOS, are indeed essential aspects of CD4^+^ and CD8^+^ T cell stimulation. While TCA cycle metabolism seems to increase after 24 h of stimulation, aerobic glycolysis increases more rapidly within six hours (53–55).

Tumor necrosis factor alpha (TNF-α), IL-12, and interferon-gamma (IFN-γ) induce metabolic rewiring of effector T cells *via* glycolytic signaling pathways, such as PI3K–AKT–mammalian target of rapamycin (mTOR) (53, 56, 57). This glycolytic transition enables rapid ATP generation, NAD^+^ renewal, and nucleotide biosynthesis, all of which are essential for effector function, cytokine secretion, and cell growth. IL-2 autocrine secretion boosts GLUT1 expression and the activation of the PI3K pathway (56, 58). When the T-cell receptor (TCR) is activated, opposing moderators of PI3K signaling (PTEN) and PI3K Interacting Protein 1 (PIK3IP1)/transmembrane inhibitor of PI3K (TrIP) are downregulated, allowing metabolic rewiring (59, 60). PI3K phosphorylates AKT, stimulating the expression of GLUT1 to convey glucose and regulate glycolytic enzymes, including LDHA, HK2, and PKM2 (54, 56). Furthermore, mTOR and c-MYC activation boost HIF-1 expression to support glycolysis and reduce oxygen consumption rate (OCR) in CD8^+^ T cells (54). As a result, the deletion of HIF in CD8+T cells lowers their penetration into cancerous tissues and, as a result, enhances tumor development (61). A current finding explored more information about the process of glycolysis as it relates to the stimulation of T cells such as CD4 + and CD8 + T cells. Several studies underlined two separate phases; the first is rapid glycolysis, which begins a few minutes after TCR activation and is controlled by pyruvate dehydrogenase kinase 1 (PDHK1), which diminishes OXPHOS but stimulates lactate generation independently of glucose intake. CD28, the PI3K axis, and HIF-1 activation will sustain glycolysis at a high rate (52).

Myeloid cells also have a function in tumor immunity (62). Macrophages, which are produced from circulatory monocytes, help to phagocytose dead cells and secrete cytokines (62). Through the major histocompatibility complex (MHC), they also can deliver antigens (tumor peptides) to naïve T lymphocytes *via* MHC molecules. Activated macrophages, such as T cells, are divided into subgroups. Whether macrophages transform into classical anticancer M1-like morphologies or an alternative suppressive M2-like phenotype is influenced by the metabolic shift that happens during infections or malignancies. The metabolic pattern will determine macrophage polarization and impact people with cancer prognosis (63, 64). TAMs (tumor-associated macrophages) have been demonstrated to exhibit an M2-like phenotype in cancer. Therefore, the oxidative metabolism of protumoral M2-like macrophages increases their proliferation at the expense of M1. M2 cells can develop their immunosuppressive phenotype by undergoing a metabolic transition in response to anti-inflammatory markers such as IL-4 and IL-10 and glucocorticoid activation. They boost their oxygen consumption rate to facilitate their expansion (65). Their oxidative metabolism is supported by an increase in the amount and content of mitochondria, as evidenced by the increased expression of succinate dehydrogenase A (SDHA) (65–67). In the setting of M2 polarization, fatty acid oxidation (FAO) is the primary energy source for the TCA cycle. The interplay between the signal transducer and activator of transcription (STAT6) and peroxisome proliferator-activated receptors (PPARs) is induced by IL-4 stimulation, which enhances CD36 expression to bind and translocate fatty acids in cells (65, 68). Many investigations have demonstrated that lipid oxidation is required for M2-like/TAM development. FAO is necessary for tumor progression, propagation, and migration *via* IL-1 production (62). Unlike M1 macrophages, which use Nitric Oxide Synthase 2 (NOS2) to create NO and destroy cancer cells, M2-TAMs metabolize arginine to polyamines through increasing Arginase 1 expression (62). Arginase 1 activity promotes cancer growth, spread, and angiogenesis and has been associated with a poor prognosis in various types of cancer (69–72). Further details are depicted in **Table 1**.

**Table 1:** Metabolic reaction of anti-tumoral and pro-tumoral immune cells in cancer.

Immune cell	Metabolic reaction	Description	Ref.
**Naive T cell**	OXPHOS, FAO	Naïve T cells produce ATP through OXPHOS and FAO controlled by the transcription factor FOXO1 and IL7 activation. T cells maintain low glycolytic activity by repressing mTOR signalization *via* Tsc1 expression.	(73, 74)
**T CD4**	Glycolysis	ThE glycolytic switch allows fast ATP supply, regeneration of NAD+ and nucleotides synthesis required for effector activity, cytokine production and cell proliferation.	(53, 56)
**T CD8**	Glycolysis	the activation of mTOR and c-MYC increases HIF-1α levels to reinforce glycolysis and decreases OCR of CD8+ T cells	(75)
**Macrophage**	Glycolysis, OXPHOS	Activation of PI3K signaling pathway and overexpression of HIF-1α in M1 macrophages promote glycolysis by upregulation of glycolytic enzymes such as GLUT1, PKM2 and HK2. The oxidative metabolism of protumoral M2-like macrophages promotes their expansion to the detriment of M1. Their oxidative metabolism is sustained by an increase in the number of mitochondria and their mitochondrial content, reflected by an upregulation of succinate dehydrogenase A (SDHA)	(65, 66, 76, 77)
**NK cell**	Glycolysis	Upon cytokine-stimulation, NK cells increase GLUT1 expression, which is consistent with the augmented glucose uptake and glycolysis that accompanies cell activation. In the TME, tumor-driven glucose restriction may reduce glycolysis of NK cells and thus impair their antitumor functions.	(78, 79)

## miRNAs and metabolic reprogramming of cancer cells

miRNAs are short non-coding RNAs that silence their target mRNAs after transcription through transcriptional silencing or mRNA degradation (80, 81). miRNA-mediated gene expression modulation can occur *via* mRNA breakdown or translational suppression. miRNAs attach to the target mRNA’s 3-untranslated region (3-UTR) *via* incorrect base-pairing and can control a wide range of genetic components simultaneously (82–84). As a result, miRNAs, such as transcriptional regulators, could function as master gene modulators and collaborate to establish gene expression profiles in cells (85). miRNAs play roles in various biochemical processes, including growth, differentiating, propagation, apoptosis, and induced pluripotent stem cells (86).

Several lines of evidence suggest that miRNAs act as a key function in metabolic activities, notably lipid and glucose metabolism, as well as amino acid biosynthesis (11). Furthermore, miRNAs may identify and modify metabolic components at the transcriptional level, which is vital in both cancer and normal cells (12). Tumor cell metabolism may be altered to avoid apoptosis and promote cell proliferation and survival. The Warburg effect, in which miRNA dysregulation leads to higher glycolysis, is the most robust metabolic phenotype found in cancer cells (13, 14). miRNAs have a role in tumor cell metabolism regulation by modulating the expression of target genes whose compounds either explicitly govern metabolic machinery or indirectly modify the transcription of metabolic enzymes, so acting as master controllers (10). This section will summarize and discuss the involvement of miRNAs in several metabolic processes in cancer (**Table 2**).

**Table 2:** miRNAs reprogrammed metabolic pathways in tumor and tumor-associated immune cells.

Cell	Study setting	miRNA	Mediator	Metabolic effector	Outcome	Ref.
**PC**	*In vitro*	miR-378a	GLUT1	glycolysis	Through distinct pathways, miR-378a inhibits glucose metabolism and lowers tumorigenesis in PCa cells; therefore, they uncover GLUT1 mRNA as a direct target of miR-378a. This study found that inhibiting GLUT1 impairs glycolysis, causing cell death.	(87)
**BC**	*In vitro* and *in vivo*	miR-34a	LDHA	glycolysis	LDHA stimulated glycolysis and cell growth. In the present research, LDHA was found to be a primary target of miR-34a.	(88)
**BC**	*In vitro* and *in vivo*	miR-140-5p	GLUT1,	glycolysis	In breast cancer, miR-140-5p was discovered to be dysregulated, and it specifically targeted GLUT1, culminating in antiglycolytic and antiangiogenic impact.	(89)
**BC**	*In vitro* and *in vivo*	miR-186-3p	EGFR	glycolysis	This research showed an innovative molecular basis of hormone therapy resistance in which the miR-186-3p/EREG axis synchronizes tamoxifen resistance and aerobic glycolysis in ER-positive breast cancer, implying that targeting miR-186-3p is an intriguing potential treatment in endocrine-resistant breast tumors.	(90)
**HCC**	*In vitro* and *in vivo*	miR-199a	HK2 and PKM2	glycolysis	These findings show a unique mechanism of malignancy energy metabolism remodeling in which HuR reduces miR-199a development, linking hypoxia to the Warburg phenomenon, and indicate a possible treatment approach that targets miR-199a to disrupt malignant aerobic glycolysis.	(91)
**HCC**	*In vitro* and *in vivo*	miR-125a	HK2	glycolysis	By targeting hexokinase HK2, hypoxia-down-regulated miR-125a controlled HCC glycolysis and tumorigenesis, adding a novel aspect to hypoxia-mediated modulation of cancer metabolism.	(92)
**CRC**	*In vitro* and *in vivo*	miR-146b-5p	PDHB	glycolysis	Upregulation of PDHB inhibited the carcinogenic impacts of miR-146b-5p on CRC cell proliferation, invasion, and metabolism.	(93)
**CRC**	*In vitro* and *in vivo*	miR-181d	CRY2 and FBXL3	glycolysis.	MiR-181d has an oncogenic function in CRC through increasing glycolysis, as well as the miR-181d/CRY2/FBXL3/c-MYC feedback loop may be a potential treatment for CRC patients.	(94)
**Colon cancer**	*In vitro*	miR-192 and miR-215	PI3K-Akt	glycolysis	SRPX2 supported colon cancer cell glycolysis through miR-192 and miR-215, both of which are suppressed by PI3K-Akt.	(95)
**Lung cancer**	*In vitro* and *in vivo*	miR-143	HK2	glycolysis	MiR-143 was discovered to be an important determining factor of tumor glycolysis by targeting HK2.	(14)
**CRC**	*In vitro* and *in vivo*	miR-23a∼27a∼24	HIF-1α	TCA	The HIF-1α-induced miR-23a27a24 cluster regulates the glucose metabolic pathway by modulating numerous metabolic networks and affecting many TCA-related genes.	(96)
**HCC**	*In vitro*	miR-205	ACSL1	fatty acid synthesis	ACSL1, which is miR-205-targeted, might contribute to aberrant lipid metabolism in liver cancer.	(97)
**Lung cancer**	*In vitro*	miR-15a-5p	ACSS2	fatty acid synthesis	This research discovered a unique method of miR-15a-5p in preventing lung cancer cell progression *via* reducing lipid metabolism *via* regulation of ACSS2-mediated acetyl-CoA activities and histone acetylation.	(98)
**Colon cancer**	*In vitro* and *in vivo*	miR‐497‐5p	acyl‐CoA synthetase‐5	fatty acid synthesis	Overexpression of miR4975p might be investigated as a treatment modality for modulating lipid metabolism in colon cancer.	(99)
**BC**	*In vitro* and *in vivo*	miR-22	ATP citrate lyase and fatty acid elongase 6	fatty acid synthesis	miR-22 was discovered to be a new modulator of tumor cell metabolism, a capability that may contribute to this miRNA’s function in cellular differentiation and cancer progression.	(100)
**Lung cancer**	*In vitro*	miR-21	CD36	fatty acid synthesis	miR-21 enhanced non-small cell lung cancer and may offer a unique treatment strategy for addressing non-small cell lung cancer in the clinic.	(101)
**Bladder cancer**	*In vitro*	miR-16	GLS2	glutamine metabolism	Urothelial carcinoma-associated 1 inhibited ROS production in bladder cancer cells *via* dealing with miR-16 and regulating GLS2 expression.	(102)
**ICC**	*In virto* and*in vivo*	miR-145	Sirt3/GDH axis	glutamine metabolism	The TUG1/miR-145/Sirt3/GDH regulatory circuit might offer a potential pharmaceutical method for ICC therapy.	(103)
**PC**	*In vitro*	mir-23	c-MYC	glutamine metabolism	In human P-493 B lymphoma cells and PC3 PC cells, the oncogenic transcription factor c-MYC drives cell propagation while transcriptionally repressing miR-23a and miR-23b, leading to increased production of its target protein, mitochondrial glutaminase.	(104)
**Ovarian cancer**	*In vitro*	miR-145	c-MYC/GLS1	glutamine metabolism	MiR-145 suppressed glutamine metabolism in ovarian cancer cells *via* the c-MYC/GLS1 pathways, which might enhance the existing cancer detection and treatment method.	(105)
**T cell**	*In vitro*	miR-143	Glut-1	glycolysis	MiR-143 improved T cell anticancer activities by increasing memory T cell development and metabolic reprogramming *via* Glut-1.	(106)
**T cell**	*In vitro* and *in vivo*	miR-101 and miR-26a	EZH2	glycolysis	EZH2 stimulated the Notch network by inhibiting Notch repressors Numb and Fbxw7 by trimethylation of histone H3 at Lys27, which boosted T cell polyfunctional cytokine production and longevity by Bcl-2 signaling.	(107)
**T cell**	*In vitro* and *in vivo*	miR-652-5p	TIGAR	glycolysis	Defective miR-652-5p/Tigar axis might suppress glycolysis, slowing T-ALL cell proliferation, establishing miR-652-5p as a unique potential clinical target for T-ALL treatments.	(108)
**T cell**	*In vitro*	miR-34a	LDHA	glycolysis	Hypoxia reduced miR-34a expression and LDHA miR-34a modulation, raising lactate levels in GC TILs and decreasing immunological activity in GC.	(109)
**MQ**	*In vitro*	miR‐30c	REDD1 and mTOR	glycolysis	Hypoxia inhibited M1 maturation and activity in the human GC microenvironment by suppressing miR30c expression and decreasing mTOR functioning and glycolysis in GC TAMs.	(110)

PC, prostate cancer; GLUT1, glucose transporter 1; BC, breast cancer; LDHA, lactate dehydrogenase A; ER, Estrogen receptor; EREG, Epiregulin; HCC, Hepatocellular carcinoma; HK2, Hexokinase 2; PKM2, pyruvate kinase M2; CRC, colorectal cancer; PDHB, Pyruvate Dehydrogenase E1 Subunit Beta; CRY2, Cryptochrome Circadian Regulator 2; FBXL3, F-Box And Leucine Rich Repeat Protein 3; PI3K, phosphoinositide 3-kinase; SRPX2, Sushi Repeat Containing Protein X-Linked 2; HIF-1α, Hypoxia-inducible factor 1-alpha; TCA, tricarboxylic acid; ACSL1, Acyl-CoA Synthetase Long Chain Family Member 1; ACSS2, Acyl-CoA Synthetase Short Chain Family Member 2; GLS2, Glutaminase 2, ROS, Reactive oxygen species; ICC, intrahepatic cholangiocarcinoma; Sirt3, Sirtuin 3; GDH, glutamate dehydrogenase; GLS1, Glutaminase-1; EZH2, Enhancer of zeste homolog 2; Notch, Neurogenic locus notch homolog protein; Numb, UMB Endocytic Adaptor Protein; Fbxw7, F-Box And WD Repeat Domain Containing 7; TIGAR, TP53-induced glycolysis and apoptosis regulator; T-ALL, T-cell acute lymphoblastic leukemia; GC, gastric cancer; TILs, Tumor-infiltrating lymphocytes; MQ, macrophage; REDD1, regulated in development and DNA damage responses 1; mTOR, mammalian target of rapamycin.

## miRNAs in glycolysis

**Figure 1:**
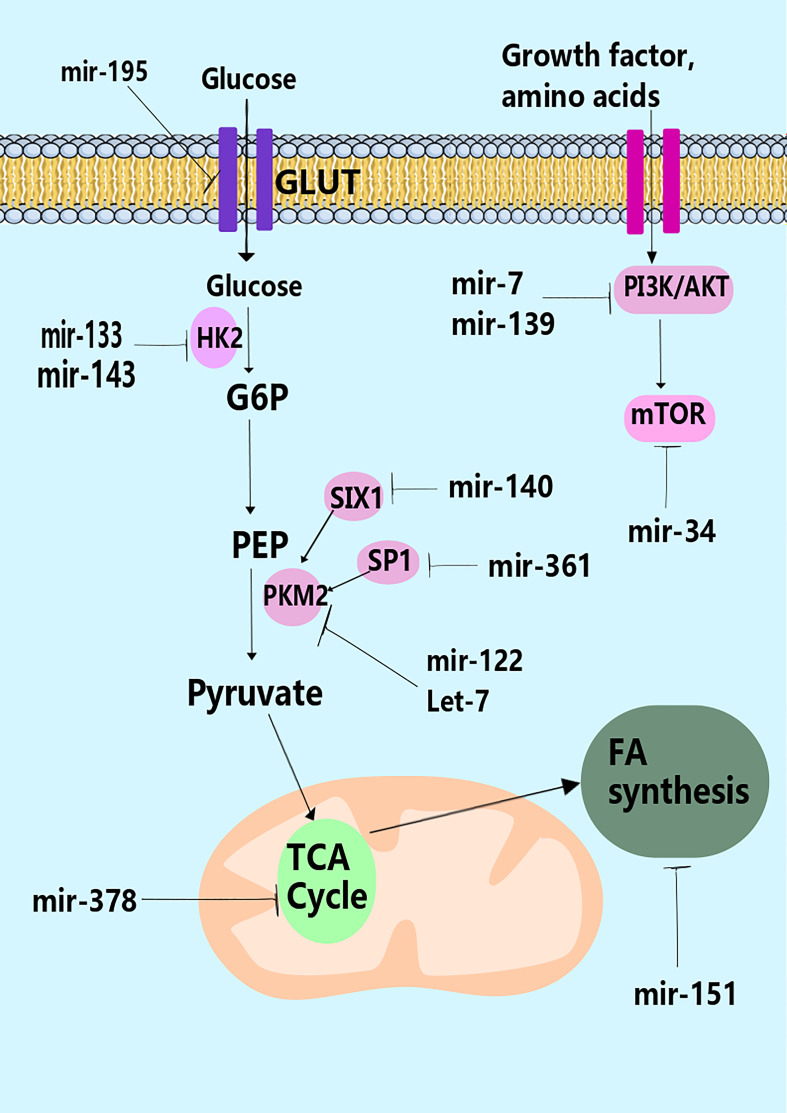
miRNAs and control of metabolism. miRNAs have been shown to regulate many stages in metabolism through modulation of the expression of key metabolic enzymes (e.g., hexokinase-2 by miR-133), transporters (e.g., GLUT by miR195-5p), or pathways that control metabolism such as the PI3K pathway (e.g., miR-19, miR-7, and miR-139).

**Figure 2:**
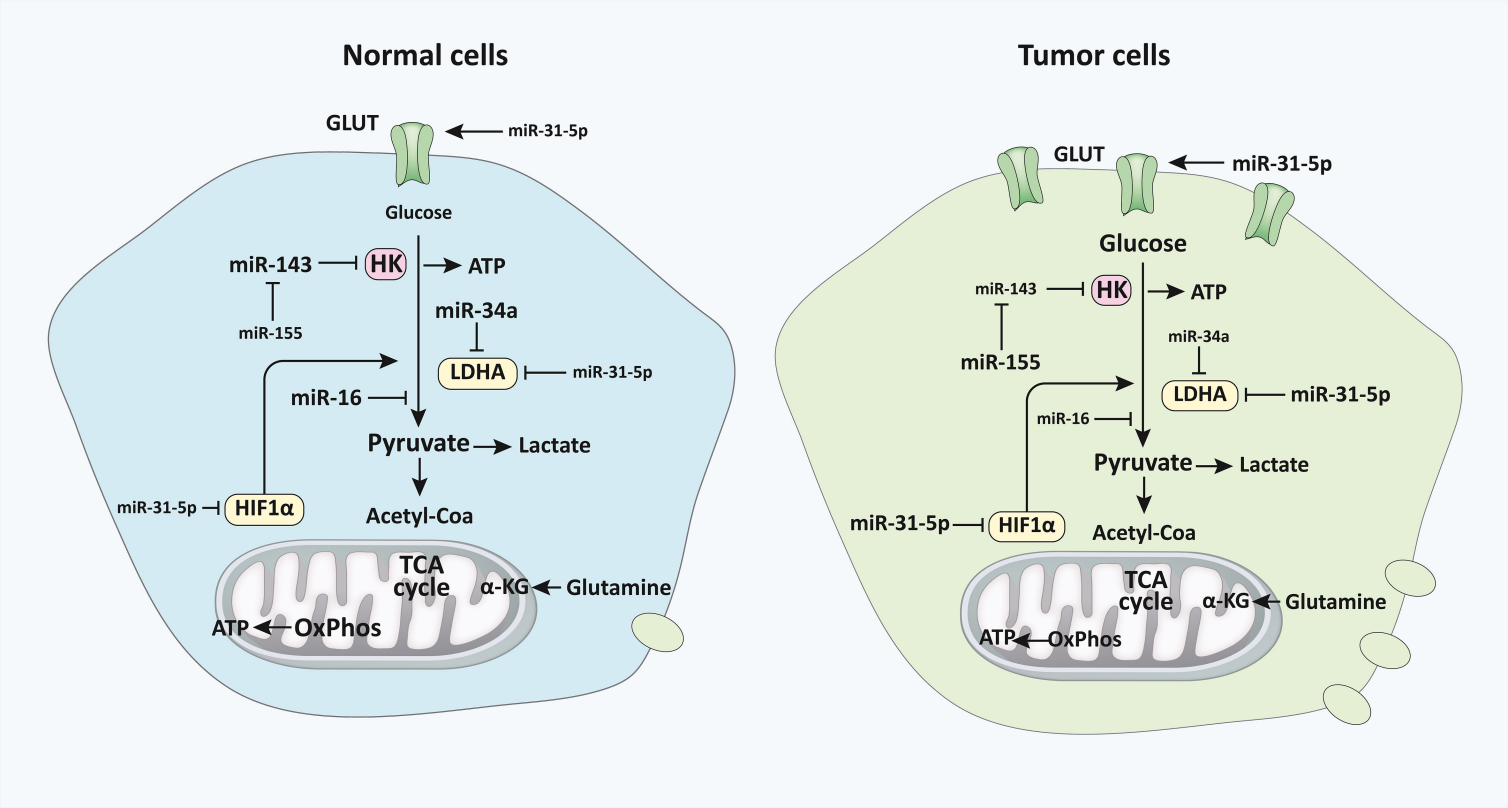
miRNAs regulate glucose metabolism in normal vs malignant cells. The diagram represents the primary alternative energy metabolism pathways in human cells and their proportionate use in normal quiescent cells and tumor and highly proliferative cells, which are depicted *via* varying degrees of clarity. The letter size distinguishes the frequency of recognized regulatory miRNAs, their primary mRNA targets (HK, LDHA, and HIF1), as well as particular indirect miRNA regulatory interplays with other miRNAs or pathway interactions. Modulatory connections are represented by referring arrowheads, whereas repressive interactions are represented by inhibiting arrows. The designations of pathways and metabolites are emphasized in bold and thicker lines to indicate their predominance in each condition (33). miRNA, microRNA**;** HK, Hexokinase; LDHA, lactate dehydrogenase A; HIF1, Hypoxia-inducible factor 1.

miRNAs can regulate metabolic pathways, several of which are altered in malignancies and other cellular activities (111). The downstream targets of several distinct miRNAs have been directly or indirectly linked to alterations in tumor metabolism. According to research, miRNAs govern the irreversible processes of glycolysis, particularly the critical enzymes (**Figures 1**, **2**) (112). For example, miR-143 controls glycolysis by addressing Hexokinase 2 (HK2), which phosphorylates glucose to create G6P, so dedicating glucose to anaerobic glycolysis (14). HK2, coding for the first rate-limiting enzyme of glycolysis, is among the top list of genes predicted and potentially regulated by multiple miRNAs, including miR-143. Besides, the miR-200 family, which includes miR-200a, miR-200b, and miR-200c, has been shown to control phosphoglucose isomerase, which is also associated with tumorigenesis (113). Current results suggest a role of miR-200s in PGI/AMF-induced EMT, and thus approaches for up-regulation of miR-200s could be a novel therapeutic strategy for the treatment of highly invasive breast cancer. Furthermore, miR-17-92 regulates the amounts of enolase (ENO) 1, ENO2, phosphoglycerate kinase 1 (PGK1), and triosephosphate isomerase 1 (114). A quantitative mass spectrometry analysis indicated that oxysterol-binding-protein-related-protein 8 (ORP8) targets miR-143 (115). miR-155 by stimulating the STAT3 (a transcriptional activator for HK2) can block miR-143 (116). Additionally, miR-143 decreases the expression of HK2 in both primary keratinocytes and cell lines generated from head and neck squamous cell carcinoma (117). Furthermore, HK2 has been identified as a miR-143 target, suggesting that miR-143 may influence glucose metabolism in colon cancer cells (118). Similarly, miR-143 has also been found as a determinant factor in cancer glycolysis in human lung cancer by addressing HK2 (14).

Through carefully fusing external stimuli with the availability of amino acids and intracellular energy, the mammalian target of rapamycin (TOR) regulates the cellular development and metabolic functions of all eukaryotic cells (119). The expression of various glycolytic enzymes, including GLUT1, LDHB, and PKM2, is increased when mTOR is activated (120, 121). In response to growth factors and PI3K/AKT signaling, the activation of mTOR signaling causes an increase in HIF-1 protein synthesis, which leads to the overexpression of glycolytic enzymes (122, 123). Some studies have found a vital function for miRNAs in controlling mTOR through PI3K/AKT-independent and dependent pathways. Nagaraja et al. (124) showed that miR-100 directly targets mTOR and actively suppresses its signaling in ovarian cancer. A negative relationship was discovered between miR-193a-3p and mTOR by Fornari et al. (125). They found that miR-193a-3p directly repressed the translation of mTOR1 and c-met in hepatocellular carcinoma, which altered the cell cycle, increased invasiveness, and made cancer cells more susceptible to doxorubicin therapy and hypoxia-induced apoptosis (125). Goa et al. (126) found that miR-126 prevents the growth of tumor cells by explicitly targeting the p85b subunit of PI3K in a different investigation. Since PI3K/AKT triggers downstream signals to mTOR for cellular proliferation, miR-126 loss increases tumor formation (126). Similarly, Pineau et al. (127) demonstrated a negative connection between miR-221 and the expression of DDIT4, a regulator of the PI3K/AKT/mTOR pathway in liver cancer cell lines. Several miRNAs might alter glucose homeostasis *via* mechanisms other than aerobic glycolysis. MiR-375 is one such miRNA that may regulate glucose homeostasis in the body *via* modulating insulin release (111). In β cells, the upregulation mentioned above limits insulin exocytosis through processes that are independent of transmembrane Ca2+ fluxes and intracellular Ca2+ signaling (128). Several earlier studies have shown that miR-375 regulates glucose homeostasis in pancreatic β cells *via* modulating the phosphatidylinositol 3-kinase (PI3K) signaling pathway (129). MiR-375 expression was significantly reduced in pancreatic cancer cells (130, 131). As a result, the precise impact of downregulation on glucose metabolism and possible targets in cancer cells has not been discovered.

## miRNAs and pentose phosphate pathway

Multiple pathways branch off from glycolysis, the first of which is the PPP (132). Because NADPH and nucleic acid precursors are required for cancer transformation in PPP, it is vital for glucose catabolic and anabolic pathways. Glycolysis can be directed to the PPP, which provides NADPH to cells while storing reduced glutathione (GSH) and ribose-5-phosphate, a substrate for nucleotide synthesis (133). Co-regulation of glycolysis and flux to the PPP is critical for tumorigenesis and/or malignancy. The conversion of G6P into PPP, in particular, enhances cancer aggressiveness by producing ribose 5-phosphate and reducing equivalents such as NADPH, which is used in lipogenesis and also mitigation of oxidative stress (134). In lung tumors, miR-1 and miR-206 mediate the increase in G6P and subsequent decrease in nuclear factor erythroid-related factor 2 (NRF2) function (135). The levels of G6PD are increased in some tumors, and the enzyme is regulated by miR-206 and miR-1 in cervical tumors associated with papillomavirus infections, while miR-1 downregulates G6PD in malignant tumors (136–138). Moreover, in HCC specimens, the amounts of G6PD are inversely correlated with miR-1 and the liver-specific miR-122, and the reduction of expression of these two miRNAs enhances tumorigenesis (52). A simultaneous decline in the expression levels of miR-122 and miR-1 is expected to contribute to the dysregulation of glucose metabolism in HCC cells, resulting in fast tumor development. It has been revealed that miR-122 depletion has a larger influence on HCC due to its prevalence in the benign liver and drastic reduction in HCC.

Different glucose metabolite patterns have been discovered in colorectal cancer (CRC) cases. A growing body of research indicates that pro-inflammatory mediators and critical rate-limiting enzymes of glycometabolic pathways may control impaired glucose metabolism in CRC, leading to distinct metabolic characteristics of cancer cells (139). However, the influence of miRNAs on glucose metabolism in CRC remained unclear. Qiu et al. (139) explored possible glycometabolism-regulating miRNAs in CRC by analyzing current miRNA expression datasets. They found miR-124 to be a potent regulator of PPP and nucleotide synthesis in CRC cells by targeting phosphoribosyl pyrophosphate synthetase 1 (PRPS1) and ribose 5-phosphate isomerase A (RPIA). Significantly, PRPS1 and RPIA, which are simultaneously elevated in CRC samples, might serve as potential markers for the poorly unknown CRC prognosis. Numerous investigations have shown that 6-Phosphogluconate dehydrogenase (6PGD) is upregulated in many malignancies (140). 6PGD has been identified as a functioning target of miR-206 and miR-613 in lung tumor cells, and miR-206 and miR-613 are thought to influence 6PGD expression and metabolic switching in cisplatin-resistant ovarian and lung tumor cells (141, 142). As a result, the uneven control of PPP and how cancer cells avoid PPP modulation constitute a new target for cancer diagnostics and therapy.

## miRNAs and TCA cycle

The TCA cycle is a common metabolic pathway in aerobic organisms. The TCA cycle is the last metabolic pathway for major nutrients (carbohydrate, lipid, and amino acid) as well as the primary hub of lipid, amino acid, and carbohydrate metabolism (143). The TCA cycle is the principal ATP generation process. It regulates energy production during mitochondrial respiration and is essential for glucose metabolism. A growing body of research has connected the primary enzyme activities of one-carbon metabolism and the TCA cycle to a wide range of malignancies (143).

Metabolic mediators are generated through glycolytic flux fuel biosynthetic pathways that create amino acids and nucleotides (144). The primary carbon source for replenishing the byproducts of the TCA cycle is glutamine. Glutaminase (GLS) converts glutamine into glutamate, which then enters the TCA cycle as α-ketoglutarate to provide energy for the cell. GLS was discovered to be a target of miR-23 (145). ATP citrate lyase (ACL) can metabolize glutamine-derived citrate to acetyl-CoA (AcCoA) and oxaloacetate (OAA). AcCoA contributes to *de novo* fatty acid (FA) production, while OAA can be converted into nucleotides and amino acids (146). These processes are responsible for producing macromolecules and organelles essential for cell growth. In cancer, several metabolic mediators have been identified; a glucose-dependent community that produces lactate and a lactate-dependent community that consumes lactate generated by neighboring cells (147). Tumor cells utilize glucose for fuel under hypoxic circumstances inside the tumor stroma and release lactate as a byproduct utilized by other cells. The hypoxia response system works by increasing the activity of glucose transporters and glycolytic enzymes (148). Let-7, miR-107, and miR-34 control glycolysis by addressing LDHA by p53 (149). Likewise, miR-181a and miR-183 could target isocitrate dehydrogenase 1/2 (IDH1/2) and TCA cycle enzymes (**Figure 1**) (150, 151). In addition, miR-378 affects the TCA cycle in breast cancer by modulating the expression of peroxisome proliferator-activated receptor-alpha, GA binding protein transcription factor, subunit (GABPA), co-activator 1-alpha (PGC-1α), and estrogen-related receptor gamma (ERRγ) (152). The regulation of PGC-1 by miR-23a may potentially assist the metabolic shift from OXPHOS to anaerobic glycolysis to produce anabolic precursors and support tumor cell growth (153). Until recently, the importance of the TCA cycle and the control of its activity was underestimated (140). Pyruvate dehydrogenase protein X component (PDHX) was shown to be a primary target of miR-26a in CRC cells, reported Chen and collaborators (154), and it is known that miR-26a may modify PDHX expression through specifically targeting the 3′UTR of PDHX. PDHX, which is found in the mitochondrial matrix, is a non-catalytic member of the PDH complex. It plays an essential role in the energy homeostasis that occurs in the mitochondria (155). Under some circumstances of aerobic metabolism, the PDH complex acts as a catalyst for the oxidative elimination of glucose and pyruvate. Of note, it has been discovered that miR-26a is responsible for regulating glucose metabolism in CRC cells. This occurs because it prevents pyruvate from converting into acetyl CoA and initiates the tricarboxylic acid cycle (154).

## miRNAs and lactate metabolism

It has recently been proven that lactate may be taken up in the TCA cycle, serve as a source of energy, and even operate as an oncometabolite with signaling capabilities (156). Lactate was previously known as a “metabolic waste product” produced by aerobic glycolysis (156). Although lactate was first recognized exclusively as a waste product of anaerobic cellular metabolism, it is now known that many cells constantly use lactate as fuel under strictly aerobic conditions (157). For instance, Wei et al. (158) demonstrated that the lactate generation produced by miR-181a leads to increased cellular proliferation. Similarly, high lactate levels have been shown to induce an aggressive phenotype in breast cancer cells (159). During aerobic glycolysis in cancer cells, most pyruvate is converted into lactate instead of AcCoA, which enters the TCA cycle in mitochondria (112). The lactate produced in the cytosol is subsequently released outside of the cells by monocarboxylate transporters (MCTs). Most of the released lactate can eventually be taken up in the liver and converted to glucose before being recycled back into tumors (112). However, lactate has also been hypothesized as an energy source for tumors or stromal cells under aerobic conditions. No known significant cancer-associated miRNAs affect LDHA, which converts pyruvate into lactate. However, miR-124, miR-29a, and miR-29b can target both human and mouse MCT1 3’-UTRs, possibly by controlling the propensity of tumor cells to release lactate (160). Among these, miR-29b is associated with a region that is frequently amplified in breast cancer, while miR-124 is widely downregulated in medulloblastoma (161). CD147, a widely expressed plasma membrane glycoprotein, regulates MCT1 and MCT4 in the plasma membrane (162). Let-7b, a tumor suppressor miRNA that inhibits cancer cell migration and invasion, as well as tumorigenesis, regulates the expression levels of CD147 (163).

## miRNAs and lipid and glutamine metabolism

Tumor cells have different lipid metabolism patterns, in which fatty acids are mainly synthesized from scratch (164). Mitochondrial function inside the pyruvate-citrate shuttle is required for fatty acid, cholesterol production, and protein acetylation. Recent advancements in current knowledge of the control of lipid metabolism reveal that miRNAs play key roles in regulating the metabolism of both cholesterol and fatty acids (165). Several miRNAs have been described to regulate lipid metabolism, including miR-122, miR-370, miR-378, miR-335, miR-125a-5p, and miR-33 (165). MiR-122 is essential in regulating cholesterol and fatty acid metabolism (166). MiR-122 has been shown to cause the formation of cholesterol-rich membrane domains and ER-associated lipid droplets (LDs). Silencing miR-122 may significantly change the balance of lipid accumulation and metabolic activity (167, 168).

Glutamine is an amino acid preferred by tumor cells, and its oxidative metabolism fuels the TCA cycle (169). It is a vital nitrogen and carbon supplier for the *de novo* generation of nucleotides, lipids, non-essential amino acids, anapleurotic intermediates in the TCA cycle, lipids, and nucleotides GSH components (40, 170). Proliferating tumor cells require high levels of glutamine (171). Many cancer cells and TME-related compounds, including miRNAs, modulate glutamine absorption and metabolism. The oncosuppressor miR-137 targets alanine, serine, cysteine, and glutamate transporter (ASCT2), negatively correlated with ASCT2 in pancreatic ductal adenocarcinoma, pancreatic cancer, glioblastoma, and CRC (134). Epigenetic downregulation of miR-137 results in increased glutamine uptake, allowing tumor cells to grow in an unfavorable microenvironment due to abundant glutamine availability (172). Upon entry, GLS converts glutamine to glutamate, an enzyme directly targeted by miR-203 and miR-153, both of which are dysregulated in melanoma and glioblastoma (173, 174). Lately, it has been shown that miR-145 inhibits glutaminolysis in ovarian cancer cells by decreasing the expression of c-MYC, which lowers GLS transcription (105). Low c-MYC levels and lower glutamine consumption might explain why miR-145 upregulation in ovarian tumors has a less proliferative capacity and is less aggressive. Also, c-Myc may direct the reprogramming of glutamine metabolism through miR-18a activation. The rate-limiting enzyme of glutathione synthesis in liver cancer is the glutamate-cysteine ligase catalytic subunit (GCLC), which is downregulated by c-Myc, lowering GSH bioavailability (175). Because active c-MYC can increase glutamine bioavailability and favor tumor cells, c-MYC/miR-18a-regulated cells are more susceptible to oxidative damage due to the lack of GSH synthesis from glutamine.

Glutamate is converted to alpha-ketoglutarate (α-KG) by glutamate dehydrogenase to fuel the TCA cycle (GDH). Transaminase enzymes, such as glutamate-oxaloacetate transaminase, can also metabolize glutamine to alpha-KG (GOT1). GOT1 activation is critical for prolonged cell growth in pancreatic ductal adenocarcinoma (176). MiR-9-5p could function as a tumor suppressor by directly targeting GOT1, reducing pancreatic cancer cell growth and metastasis while influencing glutamine-dependent NADPH generation and redox balance (177). A particular miRNA has many targets in glutamine metabolism, as it does in glucose metabolism. The depletion of miR-122, for example, causes ASCT2 and GLS to be upregulated in HCC. It has been found that ASCT2, GLS, and miR-122 can be regarded as prognostic markers (178). miR-122 levels in individuals are negatively associated with ASCT2/GLS, while upregulation of ASCT2 and GLS correlates with poor prognosis (178).

## miRNAs and metabolic regulation in immune cells during cancer

miRNAs have the potential to directly modulate several metabolic processes through various metabolic enzymes in tumor-associated immune cells and tumor-associated signaling processes, thereby influencing cancer growth (**Table 2**) (179–182). For example, hypoxia in malignant cell exosomes increases the production of some biomolecules (such as miR-let-7a), which suppresses the AKT/mTOR axis, resulting in the transition from glucose metabolism to OXPHOS metabolism (181). In TAMs, the suppression of AKT/mTOR reduces glucose catabolism and increases tumor blood vessel development (181, 183). Similarly, hypoxia impedes mTOR function in TAMs by decreasing the expression of miR-30c expression, which enhances the development, activity, and metabolic activity of M1 macrophages through targeting controlled advancement and DNA damage responses 1 (REDD1), ultimately leading to gastric cancer pathogenesis and progression (110). Various studies have shown that miRNAs mainly act on vital physiological enzymes or energetic nutrient transporters and affect the growth and development of T cells (184, 185). In this line, Zhang and colleagues (106) discovered miR-143 as a T-cell metabolic modulator that lowers glucose absorption by inhibiting the glucose receptor GLUT1. They discovered that miR-143 enhances memory T cell development and metabolic rewiring by particularly lowering glycolysis by TCR-dependent stimulation (106). This section summarizes new research on miRNAs involved in the immune regulation of cell metabolic processes in tumors.

Metabolically, naïve T cells or T cells that have not encountered their antigen rely more on OXPHOS, whereas activated cytotoxic T cells (CD8^+^) switch to the glycolytic pathway (186). Wells and colleagues (172) found let-7 miRs as essential modulators of naïve CD8+ T cell phenotypic maintenance *via* T metabolic activity in this scenario. Let-7 controls glycolysis and protein synthesis simultaneously with cell proliferation and differentiation by reducing the transcriptional activity of critical metabolic enzymes (Ldha, Pkm, Tpi, Hk2, Pfk1, and Gpd2), GLUT1, GLUT2, and the synthesis of the Yars enzyme (187). The amount of Let-7 decreases with T-cell activation, c-MYC is suppressed, and energy metabolism switches from OXPHOS to glycolysis, leading to an appropriate CTL response to virus-infected cells or cells presenting cancer antigens (188). MiR-155 is a well-studied miR involved in immunological reactions, particularly T cell responses, where it affects cytokine as well as interferon signaling through suppressor of cytokine signaling 1 (SOCS1) and STAT1 (106, 189). Recently, miR-155 has also been linked to T-cell metabolism. Using suppressing the inositol 5-phosphatase Ship1, a regulator of the mTOR pathway, Monnot and colleagues demonstrated that miR-155 increases mTOR activity and, consequently, glycolysis of CD8+ T cells and enhances T cell proliferation and effector functions (190). Exogenous induction of miR-155 in antigen-specific CD8+ T cells enhances antitumor efficacy toward low-affinity antigens in OVA-expressing melanoma cells (190). Collectively, these data support the results of Dudda and colleagues, showing that miR-155 upregulation may enhance T cell adoptive-transfer treatment in malignancy (189). Memory T cells, including CD8+ T cells and CD4+ Tregs, rely on enhanced OXPHOS and lipid oxidation rather than glycolysis, whereas cytotoxic CD8+ T cells demand glycolysis (186). Zhang and colleagues (69) demonstrated that miR-143 promotes central T memory development by blocking glycolysis by GLUT1. Notably, an immunomodulatory metabolite IDO and its derivative kynurenine, which are typically formed inside the TME, reduce miR-143 expression, showing a reciprocal link between miR-143 and metabolism (48). Tumor cells interact with activated T cells in the TME to consume resources such as glucose, which is required to sustain anaerobic glycolysis. Finally, tumor-induced glucose deprivation reduces anti-tumoral T-cell function, resulting in tumor growth (50, 107). Similarly, tumor-induced glucose deprivation increased miR-101 and miR-26a expression levels in CD8+ T cells, which leads to the downregulation of their common target, the methyltransferase EZH2, a crucial enzyme of the epigenetic polycomb repressive complex 2 (PRC2). All in all, decreased EZH2 function diminishes anti-cancer CD8^+^ T cell responses, promoting immunological subverting (107).

EZH2 is the catalytic component of the polycomb-group family that trimethylates histone H3 at Lys27 (H3K27me3) (191, 192). The restricted epigenetic signature H3K27me3 controls the transcription process in tumor cells (192). According to scientific literature, EZH2 is dedicated to developing Th1 and T helper 2 (Th2) cells in mice (193, 194). Zhao and colleagues (107) showed that EZH2 modulates effector T cell functionality and longevity in T lymphocytes. In the TME, EZH2 is an important target and detector of anaerobic glycolysis breakdown. Moreover, in T cells, researchers discovered that EZH2 transcription is regulated *via* anaerobic glycolysis metabolism through miRNAs, rendering it functional and therapeutically crucial in ovarian cancer patients (107). Considering the physiological and molecular relevance of EZH2 in polyfunctional T cells in tumors, it is critical to assess how T cell EZH2 is regulated in the TME and becomes hypoglycemic due to the Warburg effect. Significantly, Zhao and colleagues (107) discovered that primary ovarian tumor cells downregulate EZH2 expression and limit multifunctional cytokine production and lifespan of effector T cells, which may be restored by glucose feeding and 2-deoxyglucose mimetic agents. This finding shows that glycolytic metabolism regulates T cell EZH2 in the context of cancer. According to a study performed by Zhao and colleagues (107), it has been shown that the glycolytic shift affects the activation of memory T cells, multiplication, and expression of IFN-γ in animal models (24, 57, 195). Another mechanism of action research shows that cancer cells maintain high levels of miR-26a and miR-101 expression in effector T cells.

**Figure 3:**
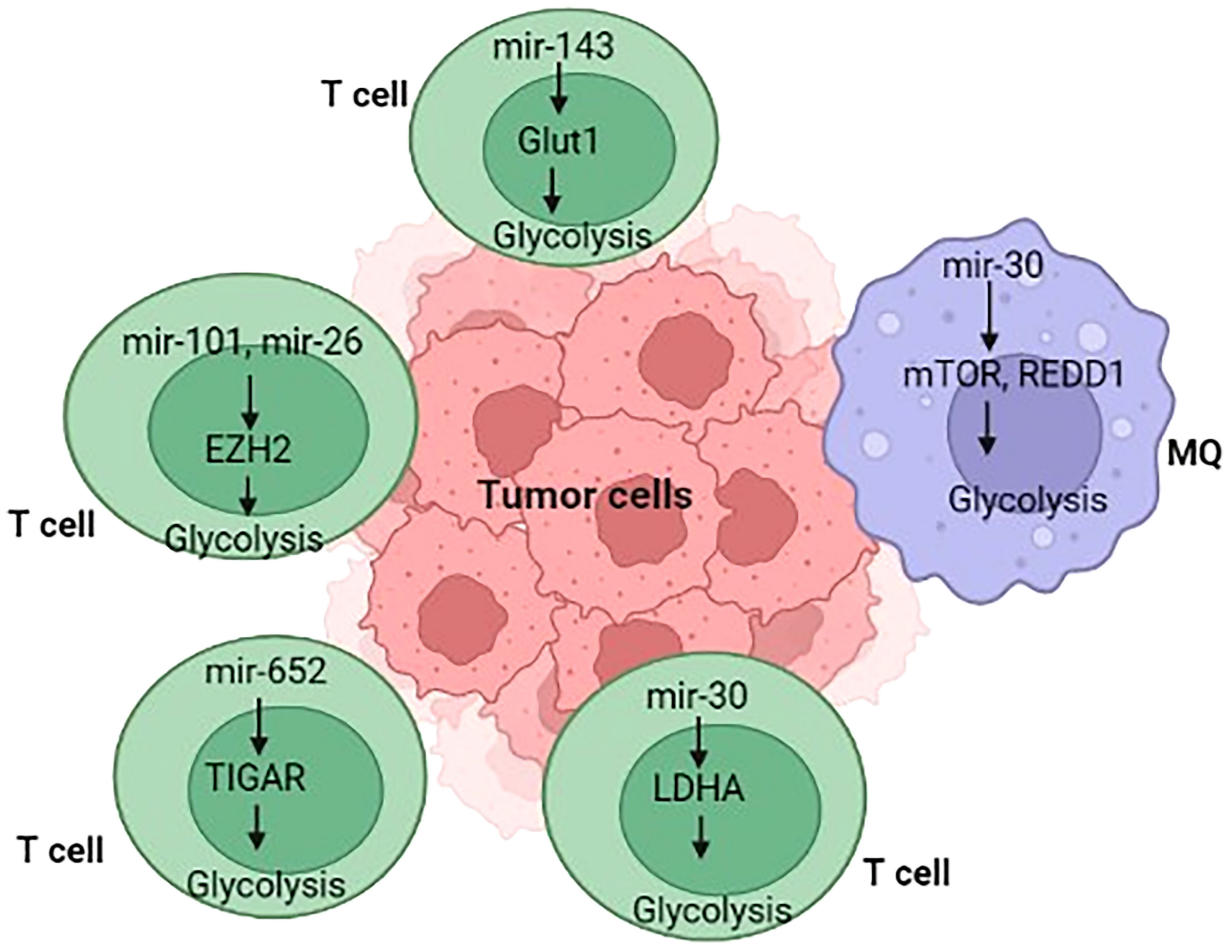
Overview of immunometabolism in tumor microenvironment.

Consequently, miR-26a and miR-101 repress EZH2 expression and impair the function of effector T cells. In conclusion, the current findings point to a metabolic target and tumor immune evasion scheme. Malignant cells inhibit T cell EZH2 activity by limiting aerobic glycolysis, reducing T cell-mediated anti-cancer immunity. miRNA expression is dynamic in naïve, memory, and effector CD8^+^ T cells throughout T cell development (196). A particular miRNA has been linked to the modulation of CD8+ T cell development. miR-17-92, for example, promotes CD8+ T memory cell growth and the formation of final effectors *via* the PI3K/AKT/mTOR axis (197). mTOR also induces the formation of HIF-1a and MYC, leading to the glucose metabolism of T cells. Nonetheless, the significance of miRNAs in the complex regulation of T metabolism in anti-cancer properties is not entirely understood. In this context, Zhang and colleagues (106) verified Glut-1 as a prominent target of miR-143 in this function to investigate the molecular mechanisms involved in enhancing the anti-proliferative activity of miR-143 in T cells. Glut-1 is the major glucose-transporter isoform in T cells, acting as a key metabolic monitoring station in glycolysis (198). Studies discovered that Glut-1 knockdown and miR-143 upregulation reduced glycolysis. A previous study has discovered that miR-143 controls tumor cell glycolysis by influencing HK2, a key glycolytic pathway enzyme (14, 199). MiR-143 was shown to suppress HK2 expression in T cells (106). T cells have a greater baseline OCR than differentiated effector cells (200). Zhang and colleagues (106) found that miR-143 upregulation resulted in greater baseline OCR and maximum respiration in T cells, which may be crucial for T cell memorization. Moreover, the dynamical expression pattern of miR-143 throughout T cell growth corresponded to the different types of energy used in naive, effector, and T memory cells (**Figure 3**). Naïve T cells create energy by breaking down amino acids, glucose, and fatty acids to fuel OxPhos. In addition, Zhang and colleagues (106) discovered that the upregulation of miR-143 increased the expression of Carnitine palmitoyltransferase I A (CPT1A). These findings suggest that miR-143 promotes Tm cell development by altering metabolic activity.

Additionally, enhanced glycolysis is observed in T-cell acute lymphoblastic leukemia (T-ALL) and is induced by oncogenes, including runt-related transcription factor 2 (RUNX2) neurogenic and locus notch homolog protein (NOTCH) (201–203). The effectiveness of glycolysis in T-ALL is reversed or reduced, suggesting that the mechanisms of glycolysis in T-ALL need to be investigated (201–203). Given that miRs have been shown to alter glycolysis levels by targeting genes encoding glycolysis rate-limiting enzymes, including phosphofructo-2-kinase/fructose-2,6-bisphosphatase 3 (PFKFB3), GLUT-1, and Phosphofructokinase-1 (PFK-1). Liu and colleagues (108) discovered that defective miR-652-5p might restrict glycolysis *via* targeting TP53-induced glycolysis and apoptosis regulator (TIGAR), explaining the decrease of PFKFB3 expression and the molecular basis of T-ALL.

Macrophages play a significant role in normal homeostasis and contribute to self-limited inflammatory response throughout infection; however, these favorable activities might be overshadowed by sustained activation signals, resulting in dysfunctional macrophage behavior, which can have pathological results (204). Like M2 macrophages, TAMs increase revascularization, reduce antigen presentation, attenuate inflammatory factor secretion, and block T-cell infiltration into the tumor (205). Before polarization, macrophages have a quiescent metabolic activity that is fuelled by the TCA cycle and depends on OXPHOS for energy (206). Master transcription regulators, including nuclear factor-κB (NF-κB) and HIF-1α, are activated by macrophage polarizing signals to modify proinflammatory gene expression and metabolic reprogramming (207, 208). Inflammatory macrophages change their metabolism programming to prefer aerobic glycolysis over OXPHOS throughout polarization (26, 209, 210). Even though aerobic glycolysis, commonly known as the Warburg effect, is less effective than OXPHOS in producing ATP, the increased glucose flow into cells results in faster energy synthesis and the creation of additional biosynthetic intermediates (211).

**Figure 4:**
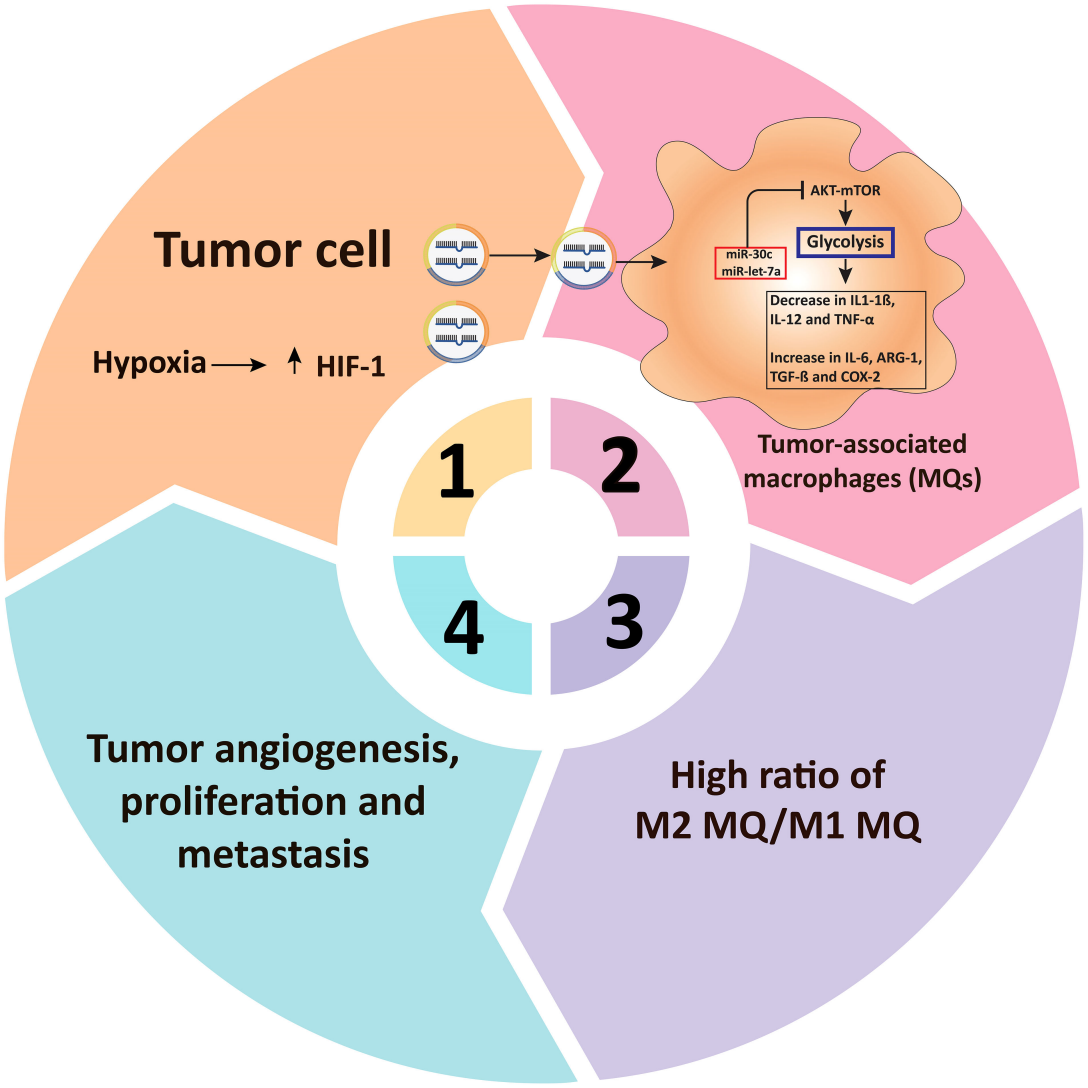
miRNAs regulate glucose metabolism in tumor-associated macrophages. In TAMs, microRNAs influence glucose metabolism. Tumor cells’ respiration is less than their oxygen demand, culminating in hypoxia as well as HIF-1 overexpression. Extracellular vesicles (including miR-30c and miR-let-7a, among several others) are secreted into TAMs through hypoxia-induced cancer cells, suppressing the glycolysis-related metabolic pathway AKT/mTOR in TAMs. Decreased glucose metabolism then raises M2-type associated factors including TGF-β, COX-2, IL-6, and ARG-1, reduces M1-type related factors including TNF-α, IL-12, and IL-1β, resulting in M2-type macrophage rewiring and tumor growth and angiogenesis (215). TAM, Tumor-associated macrophages: HIF-1α, Hypoxia-inducible factor 1-alpha; mTOR, mammalian target of rapamycin; ARG-1, Arginase 1; IL, Interleukin; COX-2, cyclooxygenase 2; TGF-β, Transforming growth factor beta; TNF-α, Tumor necrosis factor alpha.

miRNAs have been found to control several aspects of macrophage responses, such as those that increase or decrease inflammatory responses in response to polarizing stimuli (206). Recently, a large body of evidence has evolved that miRNAs are involved in modulating metabolic activities that affect macrophage activities (212). Tumor hypoxia is induced by increased metabolic rates and oxygen demand, which may contribute to the formation of persistent hypoxic conditions and the activation of HIF (213). TAMs display phenotypic characteristics similar to M2 macrophages under hypoxic circumstances (214). Hypoxia promotes the production of certain macromolecules in cancer cell exosomes, including miR-let-7a, which reduces glucose metabolism (through the associated AKT/mTOR pathway), culminating in a switch from glucose to OxPhos metabolism (**Figures 3**, **4**) (181). miRNAs also have an impact on the PI3K/AKT axis. PI3K/AKT regulates glucose metabolism by reducing PTEN function, which might increase gene expression levels in M2 macrophages, including *YM1, Fizz, ARG, TGF-β, IL-4, and IL-10* (15–17). miRNAs, including miR-32, miR-101, miR-301a-3p, and miR-103a, regulate the PI3K/AKT pathway, triggering apoptosis in cancer cells and hastening tumor formation and spread (216–219). TAMs can re-metabolize waste produced by cancer cells during metabolism, resulting in alterations in activity and phenotype. TAMs can attach to cancer cells and help them spread. Increased miR-543 expression stabilizes HIF-1 and enhances the glucose uptake ability of cancer cells (220). In cancer cells, miR-181b, miR-143, miR-4458, and miR-199a-5p enhance metastasis by boosting the function of HK2 (14, 221, 222). The downregulation of miRNAs in cancer cells, including miR-124, miR-152, miR-let-7a, and miR-139-5p, increases the expression of PKM2 expression, increasing tumor malignancy (223–226). In these situations, the glycolytic metabolite of cancer cells can induce TAMs to switch their metabolism into M2 macrophages and cooperate to accelerate tumorigenesis. Zhihua et al. (110) discovered that miR-30c promotes glycolysis and M1 polarization in human gastric cancer cells by regulating the mTOR pathway in TAMs. MiR-30c activates the PI3K/AKT/mTOR pathway by targeting the REDD1 gene, which is a negative regulator of mTOR. In cancer, hypoxia reduces miR-30c expression and decreases the proportion of anti-tumor M1 macrophages. Apart from the importance of macrophage polarization, Wenes and colleagues (227) found that TAMs lacking significant glycolytic REDD1 compete with endothelial cells for glucose consumption. Immune and tumor cells impact each other not just physiologically but by fighting for the same resources (like glucose), and they often produce miRNAs that affect metabolic pathways. Binenbaum and colleagues (228) have shown that in pancreatic adenocarcinoma, miRNA-enriched exosomes are produced by TAMs and absorbed by tumor cells, where they cause resistance to gemcitabine owing to a change in disease metabolism. While the precise mechanism is uncertain, miR-365, among other miRNAs, plays a critical role in this operation by raising the expression of pyrimidine metabolism, thus increasing the triphosphate-nucleotide pool that interferes with gemcitabine for DNA integration in tumor cells. Furthermore, miR-365 results in the activation of cytidine deaminase, an enzyme responsible for the catabolization of gemcitabine.

## miRNAs and immunometabolites and oncometabolite in cancer

Cellular metabolism influences the function of immune cells, activation, cytokine release, and anti-cancer or antiviral activity (47). A complete understanding of metabolic processes includes functional distinctions between quiescent and active TME immune cells. In this regard, metabolites (self-produced or immunometabolites), cancer cells or oncometabolite, and TME circumstances can all affect these cells (229). These substances have bioenergetic, immunological, and carcinogenic properties and can control the expression of inflammatory genes (230, 231). Succinate and citrate have pro-inflammatory effects, whereas α-KG, itaconate, and fumarate have immunosuppressive abilities (230). Most of these metabolites increase during the immune response and alter the balance between immune activation and suppression. Although high amounts of 2-Hydroxyglutarate have also been reported in cytogenetically typical malignancies, somatic mutations in cytosolic IDH1 can contribute to the development of oncometabolite 2-Hydroxyglutarate (229). IDH1 mutations were found in less than half of the individuals with elevated 2-hydroxyglutarate; the rest had mutations in IDH2, a mitochondrial homolog of IDH1. Succinate, 2-Hydroxyglutarate, and fumarate increase tumorigenesis by modulating cell signaling and influening chemotherapy and radiotherapy responsiveness *via* epigenetic processes (232, 233).

Besides, several cell metabolites, including tryptophan, are required to survive tumor-infiltrating lymphocytes (TILs) (234). Indoleamine 2,3-dioxygenase 1 (IDO1) is a tryptophan metabolism rate-limiting enzyme that converts tryptophan into kynurenine and 3-hydroxyanthranilic acid (235). IDO1 overexpression and tryptophan deficiency might lead to the dysfunction of effector T cells and tumor immune evasion (236). Upregulated miR-218 protects cervical cancer cells from immunological attack by increasing IDO1 levels (237). Lou and colleagues (238) discovered that miR-448 acts as a tumor suppressor in colon cancer cells by regulating downstream IDO1. An *in vitro* study showed that aberrant production of miR-448 is beneficial in allowing TILs to perform their full spectrum of activities (238). Huang and colleagues (239) showed that miR-153 expression is a key determinant in T cell chimeric antigen receptor (CAR) efficacy in colon cancer models. In colon cancer, miR-153 specifically targeted IDO1, enhanced CAR T cells’ cytotoxicity, and reduced tumor growth (239).

## The role of miRNAs in immunotherapy and interaction with transcription factors, as well as LncRNA/CircRNA

Recently, there has additionally been a rise in attention to deciphering the function of miRNAs in the modulation of anti-cancer immunity and how this may affect the effectiveness of various cancer therapies. This interest has been sparked by recent developments (240). Furthermore, defense mechanisms may have both pro- and anti-oncogenic effects, and the physiological communication between immune and cancerous cells in the TME is crucial in deciding how cancer will proceed. miRNAs are not only significant components of immune reaction mechanisms but also play a role in regulating and modulating a variety of interplay between immune cells and tumor cells (241).

In this context, immune checkpoint compounds are critical components in the process of modulating immunological responses (242). miRNAs, including miR-424, miR-200, miR-138–5p, miR-34a-5p, and miR-513, regulate the expression of PD-L1 and PD-1, respectively (242). Hence, the degrees of expression of these miRNAs might influence the interaction between the PD-1 receptor and the PD-L1 ligand, which in turn can modify the activities of T cells (243). MiR-138–5p is also thought to play a role in controlling some other immune checkpoint molecules known as CTLA-462. CTLA-462 is a molecule that, when it interacts with its receptors (either CD80 or CD86) on antigen-presenting cells (APC) like dendritic cells and macrophages, suppresses the function of effector T cells while simultaneously facilitating the development of regulatory T cells (Treg cells) (244). It has been shown that some miRNAs and immune checkpoint inhibitors may influence the effectiveness of other immunotherapies, including CAR T cells. miR-153 suppressed the expression of IDO in a human colon cancer xenograft cancer, which improved the impact of CAR T cells that targeted the epidermal growth factor receptor (239). Hence, to design anti-cancer therapeutic options that are both successful and tolerable and are founded on miRNAs, it is vital to properly evaluate the precise functions that miRNAs play in the TME.

Two of the most significant molecules controlling gene expression are transcription factors, or TFs, and miRNAs. At the level of transcription, TFs control the expression of genes by attaching to promoter regions, while miRNAs regulate gene expression at the post-transcriptional level by attaching to 3’ untranslated regions (245). Modulating TFs and miRNAs may either promote or inhibit tumorigenesis (246). It is significant to mention that TFs and miRNAs can control each other. They establish a feed-forward loop if they coordinate their regulation of a shared target gene (FFL). By participating in this process, major regulatory units called FFLs might continue to establish gene regulatory circuits. Aberrantly controlled TF-miRNA-mediated FFLs have been identified in various complex diseases such as CRC, glioblastoma, schizophrenia, and other diseases (245). dChip-GemiNI is the technique suggested by Yan et al. (247) to find common and particular TF-miRNA FFLs among the five different kinds of cancer. Using pan-cancer data, a more in-depth examination of TF-miRNA modulation showed 26 dysregulated FFLs across 13 different types of cancer, as well as anticipated potential genes and therapeutic targets (248).

It is well-established knowledge that non-coding RNAs, also known as ncRNAs, are responsible for a significant proportion of the human transcriptome. These non-coding RNAs mainly consist of miRNAs, long non-coding RNAs (lncRNAs), and circular RNAs (circRNAs) (249). Long non-coding RNA, also known as lncRNA, is a category of non-coding RNA with more than 200 nucleotides. According to several research findings, lncRNAs contribute significantly to the process of cancer formation in a number of different ways (250). Newly found non-coding RNA (ncRNA) with a structure consisting of a closed loop is called circular RNA (circRNA). Compared to the conventional form of linear RNA, circRNA is much more persistent because it is less susceptible to degradation by RNA exonuclease since it lacks terminal 5’ caps and 3’ polyadenylated tails (251). In cancer, it has been shown that CircRNAs can affect cellular metabolism regulation (252). Over the last several years, an ever-increasing number of scientists have committed their efforts to investigate ncRNAs’ biological activities. In addition, various computational algorithms have been created to forecast possible connections between ncRNAs and illness. This is something that is of paramount significance for the process of identifying biomarkers (253, 254).

In 2011, Salmena et al. (255) were the pioneers who proposed the competitive endogenous RNA (ceRNA) concept for the first time. A family of non-coding RNAs known as ceRNA can bind common miRNAs in a manner that competes and cross-regulates one another at the post-transcriptional stage (256, 257). This regulatory system, which is dependent on ceRNA, was found in several different malignancies. For instance, HER2 expression was controlled by the lncRNA HOTAIR thanks to its ability to compete for the miR-331-3p. This ultimately led to the formation of cancer (258). LncRNA H19 was shown to contribute to the development of oncogenic activities in gallbladder cancer through the alteration of miR-342-3p and FOXM1 (259). Additionally, circRNA ciRS-7 has the potential to behave as a “super sponge” for miR-7 and suppress the action of miR-7 (260). An increasing body of data suggests that the ceRNA regulatory network plays a role in the biological processes that contribute to the growth of HCC. These processes include propagation, dissemination, EMT, and chemotherapy resistance (261–264).

## Conclusion and perspective remarks

Throughout this overview, we emphasized that miRNAs play important roles in various fundamental processes such as immunity, gene regulation, and metabolism, especially in immune cells responding to cancer. We described the primary metabolic pathways used to fuel eukaryotic cells and the signaling networks and molecules that feed them, emphasizing the points at which they are known to be regulated by miRNAs. It has been shown how these processes are changed during the metabolic activity of tumors and how this affects immune cells invading the TME. The control of CD8^+^ T cell development has been ascribed to single miRNAs. For example, MiR-17-92 stimulates CD8+ T memory proliferation, differentiation, and terminal effector development through the PI3K/AKT/mTOR axis. mTOR additionally promotes the production of HIF-1a and MYC, leading to T-cell glucose metabolism. Notably, in human gastric cancer, miR-30c increased glycolysis and, consequently, M1 polarization through modulating the mTOR pathway in TAMs. MiR-30c activates the PI3K/AKT/mTOR axis in these cells by targeting the REDD1 gene, which is a potent inhibitor of mTOR. However, little data on the importance of miRNA-mediated metabolic wiring in immune cells are currently available in the cancer context. Unquestionably, a better knowledge of miRNA-mediated metabolic wiring of immune cells will provide new insights into the causes of immunologically related cancer and open new avenues for developing novel anticancer therapies. For example, by altering the profiles of miRNAs that target genes for critical metabolic enzymes or necessary nutrient transporters, the intracellular metabolism of immune cells can be manipulated, potentially altering metabolic rewiring and modifying immune functions. Overall, considering the importance of the regulatory role of miRNA in changing the metabolic profile of immune cells during cancer (influence on cancer fate and immune responses), future research will elucidate the precise role of these emerging regulatory elements in cancer for the discovery and potential development of novel therapeutic approaches for cancer treatment.

## Author contributions

SA, YI, AJ, AAA, HA and RZ have the idea for and planned the study and contributed to the writing of the paper. GG, AR-C, AbAA, and QQ contributed to the writing of the manuscript. SK and RM contributed to the critical revision of the report. All authors contributed to the article and approved the submitted version.

## Conflict of interest

The authors declare that the research was conducted in the absence of any commercial or financial relationships that could be construed as a potential conflict of interest.

## Publisher’s note

All claims expressed in this article are solely those of the authors and do not necessarily represent those of their affiliated organizations, or those of the publisher, the editors and the reviewers. Any product that may be evaluated in this article, or claim that may be made by its manufacturer, is not guaranteed or endorsed by the publisher.
